# Assessment of Critical Feeding Tube Malpositions on Radiographs Using Deep Learning

**DOI:** 10.1007/s10278-019-00229-9

**Published:** 2019-05-09

**Authors:** Varun Singh, Varun Danda, Richard Gorniak, Adam Flanders, Paras Lakhani

**Affiliations:** 1 Department of Radiology, Thomas Jefferson University, Philadelphia, PA 19107 USA; 2 Philadelphia, USA

**Keywords:** Machine learning, Deep learning, Artificial ntelligence, Chest radiography

## Abstract

Assess the efficacy of deep convolutional neural networks (DCNNs) in detection of critical enteric feeding tube malpositions on radiographs. 5475 de-identified HIPAA compliant frontal view chest and abdominal radiographs were obtained, consisting of 174 x-rays of bronchial insertions and 5301 non-critical radiographs, including normal course, normal chest, and normal abdominal x-rays. The ground-truth classification for enteric feeding tube placement was performed by two board-certified radiologists. Untrained and pretrained deep convolutional neural network models for Inception V3, ResNet50, and DenseNet 121 were each employed. The radiographs were fed into each deep convolutional neural network, which included untrained and pretrained models. The Tensorflow framework was used for Inception V3, ResNet50, and DenseNet. Images were split into training (4745), validation (630), and test (100). Both real-time and preprocessing image augmentation strategies were performed. Receiver operating characteristic (ROC) and area under the curve (AUC) on the test data were used to assess the models. Statistical differences among the AUCs were obtained. *p* < 0.05 was considered statistically significant. The pretrained Inception V3, which had an AUC of 0.87 (95 CI; 0.80–0.94), performed statistically significantly better (*p* < .001) than the untrained Inception V3, with an AUC of 0.60 (95 CI; 0.52–0.68). The pretrained Inception V3 also had the highest AUC overall, as compared with ResNet50 and DenseNet121, with AUC values ranging from 0.82 to 0.85. Each pretrained network outperformed its untrained counterpart. (*p* < 0.05). Deep learning demonstrates promise in differentiating critical vs. non-critical placement with an AUC of 0.87. Pretrained networks outperformed untrained ones in all cases. DCNNs may allow for more rapid identification and communication of critical feeding tube malpositions.

## Purpose

Clinicians employ enteral nutrition (EN) by feeding tubes as the primary method of nutritional supplementation for critically ill patients unable to feed themselves. Nasogastric or nasoenteric feeding tubes preserve the integrity of the intestinal microvilli and decrease the risk of bacterial transfection and thrombotic events associated with parenteral nutrition [1]. A malpositioned feeding tube in a mainstem bronchus of the lung presents with possible tracheopleuropulmonary complications including pneumonia, pleural effusions, respiratory failure, bronchopleural or pleurocutaneous fistulae, empyema, and death [2]. Thus, nasoenteric feeding tube placement is commonly confirmed by radiography after insertion and before the commencement of tube feeding. Many protocols for confirmation of nasoenteric tube placement include both chest x-ray (CXR) and abdominal x-ray (AXR) [3].

Radiologists are then responsible for accurately identifying the presence and placement of enteric feeding tubes and precluding the severe consequences associated with bronchial insertions. Because clinical demands often delay the review of these radiographs until hours after the studies are performed, a computer-aided detection (CAD) system that could expedite detection of critical results and triage patient care appropriately would be invaluable. In the past, conventional computer-aided detection (CAD) solutions often required hand-engineered rules, significant image-preprocessing and feature extraction [4]. For example, one CAD study achieved approximately 84% sensitivity for feeding tube position on radiography, but with lower specificity with up to 0.02 false positives per image, limiting its suitability for clinical use [5]. Recent significant advances in artificial intelligence using deep learning to classify images using multi-layered neural networks make an automated solution for nasoenteric feeding tube placement detection possible [6–8]. In the ImageNet Large Scale Visual Recognition Challenge (ILSVRC), all of the solutions since 2012 have used Deep Convolutional Neural Networks (DCNNs) [9]. More recently, the error rate of the best deep neural networks (< 4% percent) has exceeded that of human performance (error rate ~ 5%) [10]. A prior study evaluated the efficacy of DCNNs in the detection of endotracheal tube presence and positions on radiography [11]. However, there has not been a study evaluating the efficacy of DCNNs in classification of enteric feeding tubes on radiography. Thus, the primary goal of this study is to assess the efficacy of deep convolutional neural networks in the classification of nasoenteric feeding tube position on radiography, and specifically to distinguish between a critical bronchial insertion and a non-critical placement.

## Methods

The Tensorflow framework (Tensorflow 1.4, Google LLC, Mountain View, CA) and the Keras library (Keras v 2.12, https://keras.io) were used for training all networks in study. Naive and pretrained deep convolutional neural network models for Inception V3, ResNet50 and DenseNet 121 were each employed. The pretrained models leveraged training on 1.2 million color images (from ImageNet) while the naive models did not undergo any pretraining. 5475 de-identified HIPPA compliant radiographs were collected from the (institution blinded) picture archiving and communication system (PACS), composed of 5301 non-critical insertions (1314 with the tip in the duodenum, 707 with tip in the esophagus, 1350 with tip in the stomach, 355 normal abdominal x-rays, 300 normal chest x-rays, and 1275 normal course with the tip out of view), and 174 critical insertions (61 left and 113 right bronchial insertions). Two board-certified radiologists performed the ground-truth classifications. Images were augmented to mitigate model overfitting. Image preprocessing techniques consisted of horizontal and vertical translations, rotations (± 10 degrees), shear, and horizontal flipping. The images were split into training (4745 images), validation (630 images), and test (100 images: 50 bronchial insertions and 50 non-critical placements). The images were partitioned as such to provide sufficient data for training and enough images to validate model selection and obtain reasonable confidence intervals when evaluating model accuracy on test cases. A dropout rate of 0.5 (50%) was used in the final fully connected layers for regularization. Because there were far more images of non-critical than critical placements, oversampling was performed for critical placements.

Each architecture was used as a binary model used to distinguish between critical and non-critical findings. The top fully connected layers of the pretrained network were set to random initialization. For test cases, receiver operating characteristic (ROC), area under the curves (AUC), and 95% confidence intervals were calculated using the “exact” Clopper-Pearson method. Statistical significance of the ROC curves was assessed using a non-parametric approach using the PROC package within the R programming language (R foundation, Vienna, Austria).

## Results

**Fig. 1:**
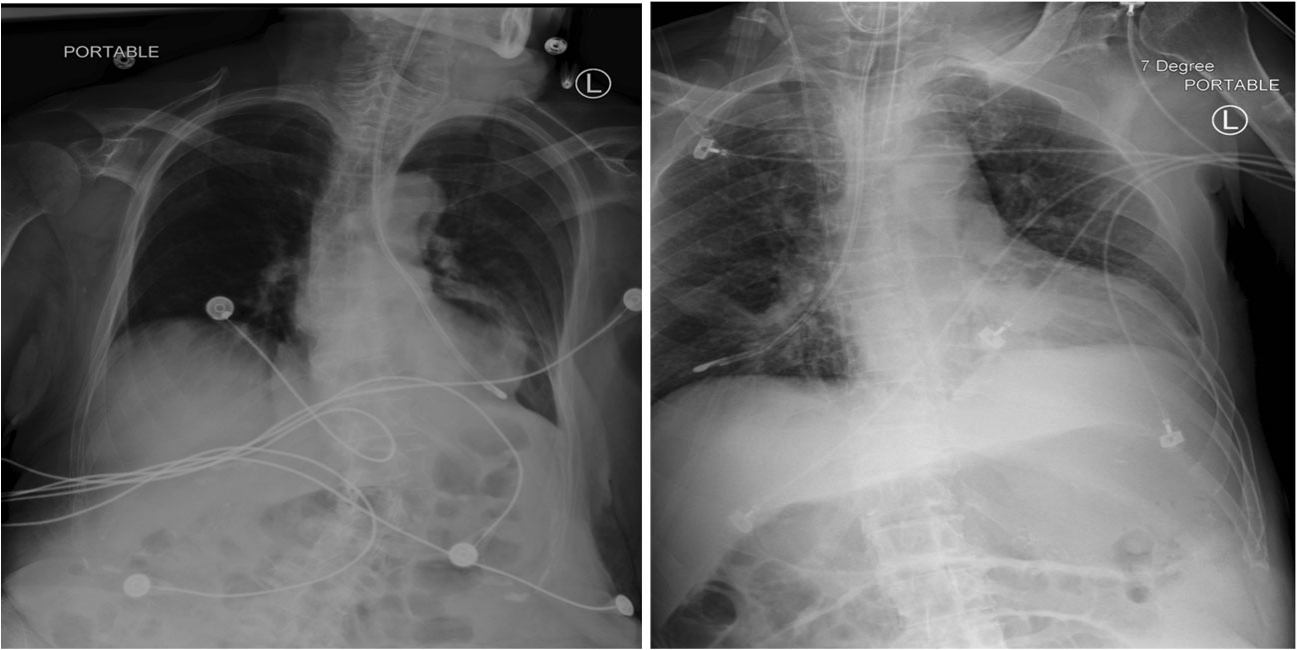
Left bronchial insertion (left) and right bronchial insertion (right)

**Fig. 2:**
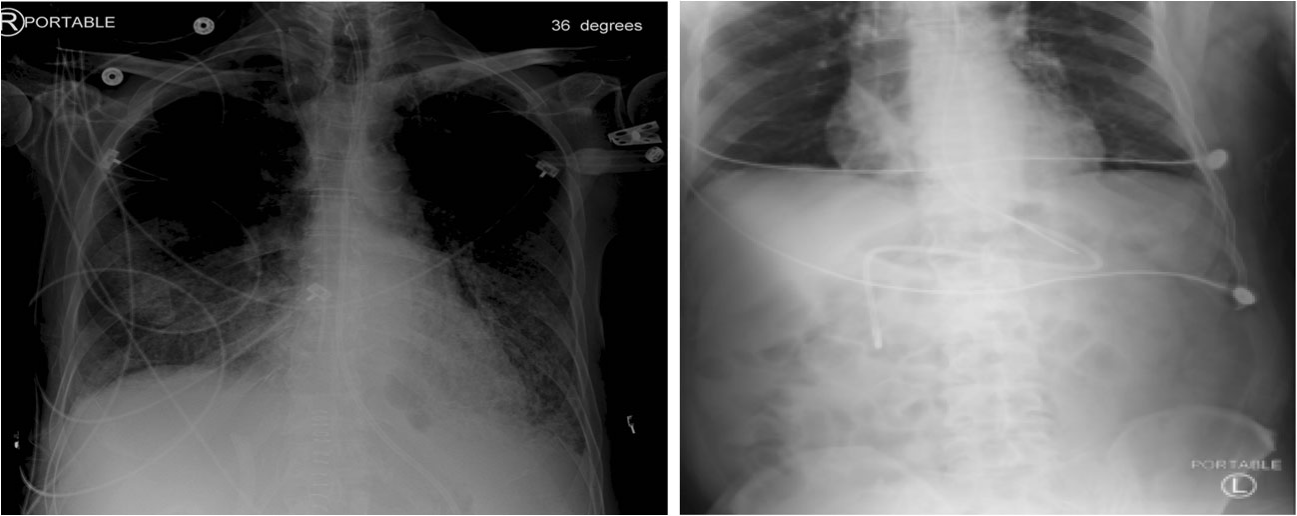
Tube normal courses with tip out of view (left) and duodenum (right)

**Fig. 3:**
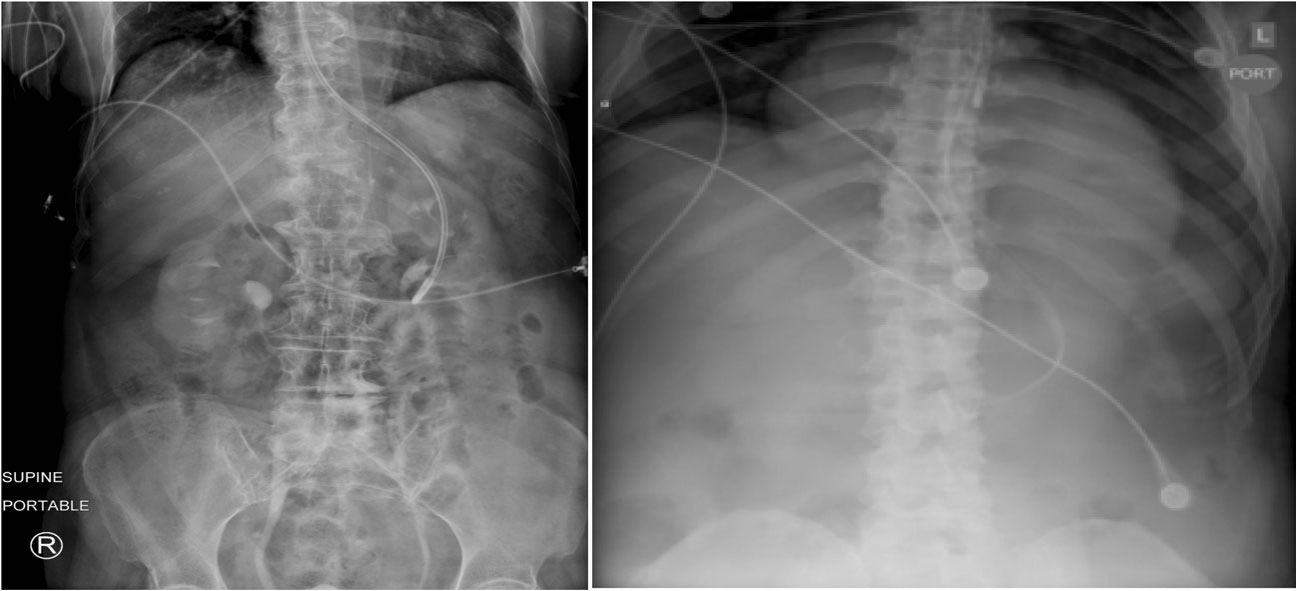
Tube placement in stomach (left) and esophagus (right)

In Table 1, for the holdout test dataset for binary classification between critical and non-critical feeding tube placement between pretrained and naive networks, the pretrained networks of Inception V3, ResNet50, and DenseNet121 outperformed each corresponding naive model. The pretrained Inception V3 had an AUC of 0.87 (95 CI; 0.80–0.94), statistically significantly greater than the naive model AUC of .60 (95 CI; 0.52–0.68) (*p* < 0.001). The pretrained ResNet50 had an AUC of 0.82 (95 CI; 0.75–0.89), statistically significantly greater than the naive model AUC of 0.60 (95 CI; 0.48–0.71) (*p* < 0.001). The pretrained DenseNet121 had an AUC of 0.85 (95 CI; 0.77–0.92), statistically significantly greater than the naive model AUC of 0.51 (95 CI; 0.45–0.58) (*p* < 0.001). There were no statistically significant differences among the AUC values between tested pretrained architectures. The pretrained ResNet model outperformed the other models by way of sensitivity with a value of 100% (95 CI; 93–100). The pretrained Inception V3 and DenseNet 121 models demonstrate higher specificities of 76% (95 CI; 62–87) and 74% (95 CI; 60–85), respectively (Figures 1, 2 and 3).

**Table 1:** Results

Network	Naive AUC	Pretrained AUC	Significance	Sensitivity (pretrained)	Specificity (pretrained)
Inception V3	0.60 (0.52–0.68)	0.87 (0.80–0.94)	*p* < 0.001	88 (76–95)	76 (62–87)
ResNet50	0.60 (0.48–0.71)	0.82 (0.75–0.89)	*p* < 0.001	100 (93–100)	62 (47–75)
DenseNet121	0.51 (0.45–0.58)	0.85 (0.77–0.92)	*p* < 0.001	92 (81–98)	74 (60–85)

Numbers in the parenthesis represent the 95% confidence interval

## Discussion

Nasoenteric feeding tube placement must be confirmed prior to the commencement of tube feeding to subvert the catastrophic complications of bronchial or esophageal placement, which include aspiration, pneumonia, respiratory failure, pulmonary fistula formation, empyema, and death [1, 12]. Radiologists are entrusted with the imperative radiographic confirmation of tube placement and the prevention of possible complications of tube malposition, but are often delayed in their review of these high-volume studies due to clinical workflow demands. A concerted human-machine approach with a validated, accurate network classifier to triage and prioritize critical findings for radiologist review could improve the detection time of bronchial insertions and clinical workflow.

Inception V3 demonstrated an AUC of .87, outperforming DenseNet121 and ResNet50, with respective AUC values of .85 and .82, although this was not statistically significant. The most sensitive model was ResNet50, which was 100% sensitive but only 62% specific. While a triage tool often demands high sensitivity at the expense of lower specificity, a model greater specificity is also important, since a high number of false positives can mitigate the efficacy of such algorithms, particularly given the relatively high-volume portable chest radiographs in most hospitals. Cascading, or the use of an ensemble of a high-sensitivity network followed by a subsequent high-specificity network, represents a potential strategy to improve statistical performance and clinical applicability [13].

**Fig. 4:**
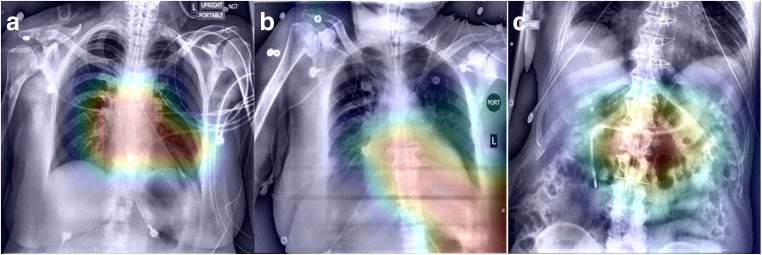
Class activation maps (CAMs) of correct class predictions. **a** Left bronchus. **b** Tip out of view. **c** Duodenum

**Fig. 5:**
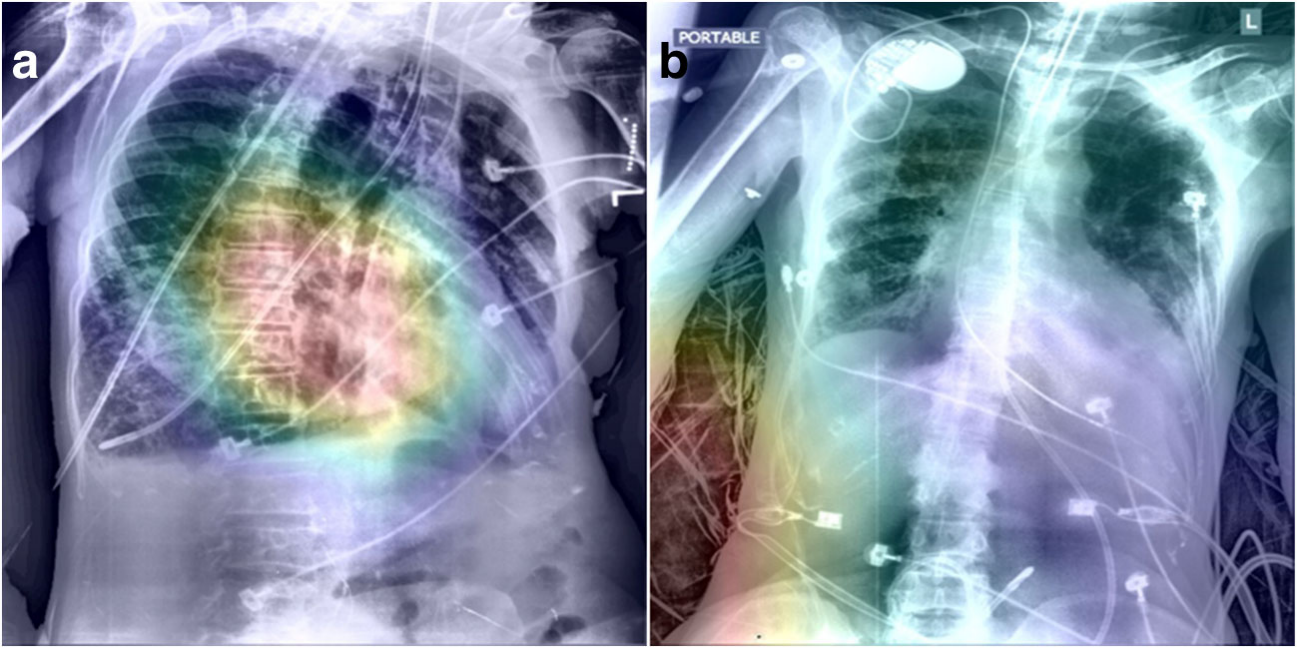
Class activation maps (CAMs) of incorrect class predictions. **a** Right bronchus. **b** Tip out of view

Figures 4 and 5 represent class activation maps (CAMs) from the Inception V3 network. CAMs are determined from the final convolutional layer of the neural network through direct visualization of the predicted class scores and are utilized to identify features most relevant to the prediction class [14]. Figure 4 demonstrates correct class predictions from the Inception V3 model, including the predictions of critical (4A, left bronchus), non-critical (4B, normal course, tip out-of-view), and non-critical (4C, duodenum). The CAMs in Fig. 4 demonstrate appropriate fitting of the network to features of the feeding tube and accurate class predictions.

Figure 5 demonstrates incorrect class predictions from the Inception V3 model, including a non-critical prediction for a critical right bronchial insertion (5A) and a critical prediction for a non-critical tip out of view (5B). In Fig. 5a, the CAM demonstrates that the network is incorporating the features of the right bronchial insertion into its class prediction, but erroneously and unaccountably predicts a non-critical placement. It is possible that the network in this case may have been negatively affected by the extreme patient rotation in the image. The CAM in Fig. 5b demonstrates that the network is incorporating features of the radiograph not relevant to tube position into its prediction class and a critical prediction for a benign tube placement. One possible explanation for the erroneous prediction in Fig. 5b is model fitting to the patient’s blanket present in the radiograph, which represents a feature not characterized or underrepresented in the training dataset. The integration of a companion model with object detection outputs and saliency features could potentially focus network predictions towards the tube region of interest and away from irrelevant features of the radiograph. Direct feeding tube object detection outputs would also direct radiologists and clinicians to exact tube position in both critical and non-critical predictions. Another potential method to improve accuracy of the model is to first segment the mediastinum and central airways using a deep learning approach, followed by a classification model.

The smaller datasets accessible in medical imaging impose the risk of model overfitting, which results in an inaccurate classifier generalizing poorly to test datasets and novel radiographs. Dropout for regularization was a major strategy used in this study to mitigate model overfitting. Another strategy that was employed to combat overfitting including augmentation of the images using various transformations, such as translation, sheer, rotation, and horizontal flipping.

DCNNs provide an encouraging solution in the binary classification of critical vs. non-critical tube placement with an AUC of 0.87 to automate the prevention of the devastating consequences associated with feeding tube malpositions. Other ways to improve the feeding tube placement classifier include using other neural network architectures, ensembling multiple deep convolutional neural networks, acquiring a larger dataset, and employing strategic preprocessing techniques well-suited to assist DCNNs in radiographic feature extraction.

## Conclusion

Deep learning demonstrates promise in differentiating critical from non-critical placement with an AUC of 0.87. Increases in training data set size, airway and mediastinal segmentation, and incorporation of companion DCNNs hold potential in improving the performance of future models.
